# Next Generation Driver for Attosecond and Laser-plasma Physics

**DOI:** 10.1038/s41598-017-05082-w

**Published:** 2017-07-12

**Authors:** D. E. Rivas, A. Borot, D. E. Cardenas, G. Marcus, X. Gu, D. Herrmann, J. Xu, J. Tan, D. Kormin, G. Ma, W. Dallari, G. D. Tsakiris, I. B. Földes, S.-w. Chou, M. Weidman, B. Bergues, T. Wittmann, H. Schröder, P. Tzallas, D. Charalambidis, O. Razskazovskaya, V. Pervak, F. Krausz, L. Veisz

**Affiliations:** 1 Max-Planck-Institut für Quantenoptik, Hans-Kopfermann Strasse 1, 85748 Garching, Germany; 2 Ludwig-Maximilian-Universität München, Am Couloumbwall 1, 85748 Garching, Germany; 3 ICFO - The Institute of Photonic Sciences, Av. Carl Friedrich Gauss, 3, 08860 Castelldefels (Barcelona), Spain; 4 Service des Photons, Atomes et Molécules, CEA, DSM/IRAMIS, CEN Saclay, 91191 Gif-sur-Yvette, France; 5 Department of Applied Physics, Benin School of Engineering and Computer Science, Hebrew University of Jerusalem, Jerusalem, 91904 Israel; 6 State Key Laboratory of High Field Laser Physics, Shanghai Institute of Optics and Fine Mechanics (SIOM), Chinese Academy of Sciences (CAS), P. O. Box 800-211, Shanghai, 201800 China; 7 Peking University Shenzhen SOC Key Laboratory, PKU-HKUST Shenzhen-Hong Kong Institution, Shenzhen, 518057 China; 8 Wigner Research Centre for Physics, Hungarian Academy of Sciences, Association EURATOM HAS, Budapest, Hungary; 9 Foundation for Research and Technology-Hellas, Institute of Electronic Structure and Laser, PO Box 1527, GR-711 10 Heraklion Crete, Greece; 10 Department of Physics, Umeå University, SE-901 87 Umeå, Sweden

## Abstract

The observation and manipulation of electron dynamics in matter call for attosecond light pulses, routinely available from high-order harmonic generation driven by few-femtosecond lasers. However, the energy limitation of these lasers supports only weak sources and correspondingly linear attosecond studies. Here we report on an optical parametric synthesizer designed for nonlinear attosecond optics and relativistic laser-plasma physics. This synthesizer uniquely combines ultra-relativistic focused intensities of about 10^20^ W/cm^2^ with a pulse duration of sub-two carrier-wave cycles. The coherent combination of two sequentially amplified and complementary spectral ranges yields sub-5-fs pulses with multi-TW peak power. The application of this source allows the generation of a broad spectral continuum at 100-eV photon energy in gases as well as high-order harmonics in relativistic plasmas. Unprecedented spatio-temporal confinement of light now permits the investigation of electric-field-driven electron phenomena in the relativistic regime and ultimately the rise of next-generation intense isolated attosecond sources.

## Introduction

The development and proliferation of intense lasers with sub-two optical-cycle duration during the past decade has allowed to create the tools and techniques for the observation and control of electronic motions in all forms of matter; a field nowadays known as attosecond physics^1^. These techniques have meanwhile provided direct time-domain access to a wide range of electron phenomena with a sub-fs resolution, such as miniscule delays in photo-emission timing^2, 3^, charge migration in molecules^4, 5^ and solids^6, 7^, as well as collective electron motion in extreme laser-plasma interactions^8^.

Powerful few-cycle laser pulses have traditionally been produced via chirped-pulse amplification (CPA) in titanium-doped sapphire (Ti:Sa) in conjunction with spectral broadening in gas-filled hollow-core fibres (HCF)^9^. CPA-based lasers have achieved peak powers beyond 1 PW, but only with pulse durations extending to about ten optical cycles or longer^10, 11^. Spectral broadening in HCFs provides octave-spanning spectra, but the approach is still limited to pulses with a few millijoules in energy^12, 13^.

Due to these restrictions few-cycle-driven attosecond sources based on high-harmonic generation (HHG) in gas targets generally suffer from a low intensity, constituting a major limitation to pushing the frontiers of the field. Upscaling few-cycle-driven HHG to higher driving pulse energies^14–16^ allows the generation of intense isolated attosecond pulses for time-resolved nonlinear optics experiments in the extreme-ultraviolet (XUV) spectral region and beyond^17–19^. Moreover, the energy scaling of few-cycle technology offers unique spatio-temporal confinement of the laser energy^20^, opening the door for a novel regime of relativistic interactions^21–23^, unprecedented control of ions^24^, as well as the generation of attosecond electron and photon bunches^21, 25–30^.

Among the various attempts at scaling the laser pulse energy while maintaining a broad bandwidth, noncollinear optical parametric chirped-pulse amplification (NOPA) is one of the most promising approaches^31, 32^. This technique transfers energy to a stretched seed from a pump pulse propagating through a nonlinear crystal in a noncollinear geometry. NOPA provides much broader gain bandwidth and much higher single-pass gain than conventional lasers^33^ as well as a cleaner temporal structure [temporal contrast, see methods] due to the short temporal gain window defined by the pump pulse duration. However, the achieved gain bandwidths proved to be insufficient to support sub-two-cycle pulse duration due to phase-matching limitations^34–38^.

Recent developments have led to the concept of enhancing the amplification bandwidth by separately amplifying and controlling the phase of the various spectral regions^39^, which we briefly refer to as optical parametric synthesis/synthesizer (OPS). This can be implemented in several ways: sequentially^40, 41^, in a parallel architecture^42, 43^, or in the Fourier-domain^44^. The concept offers the potential for energy scaling to tens of millijoules or higher. This scaling and hence its implication for next-generation attosecond sources could not be demonstrated until now.

Here, we present the first successful energy scaling and application of the sequential-OPS concept^40^. By utilizing this technique, the spectrum of the seed is amplified and coherently synthesized from two complementary NOPA stages to cover a spectral range approaching a full octave (580–1020 nm). We experimentally demonstrate that spatio-temporal focusing close to diffraction and bandwidth limit along with superior temporal contrast allow the implementation of most-demanding experiments involving field-matter interactions at relativistic intensities in solid-density plasmas. The novel OPS source, which we dubbed Light Wave Synthesizer 20 (LWS-20), provides ultrahigh-contrast, sub-two-cycle pulses with a current peak power of 16 TW focusable to peak intensities beyond 10^20^ W/cm^2^ (normalized vector potential >6). Extended with a single-shot carrier-envelope-phase (CEP) meter, the LWS-20 provides few-cycle fields for relativistic interactions with a known evolution of the driving electromagnetic fields for the first time.

## The Light Wave Synthesizer 20

**Figure 1:**
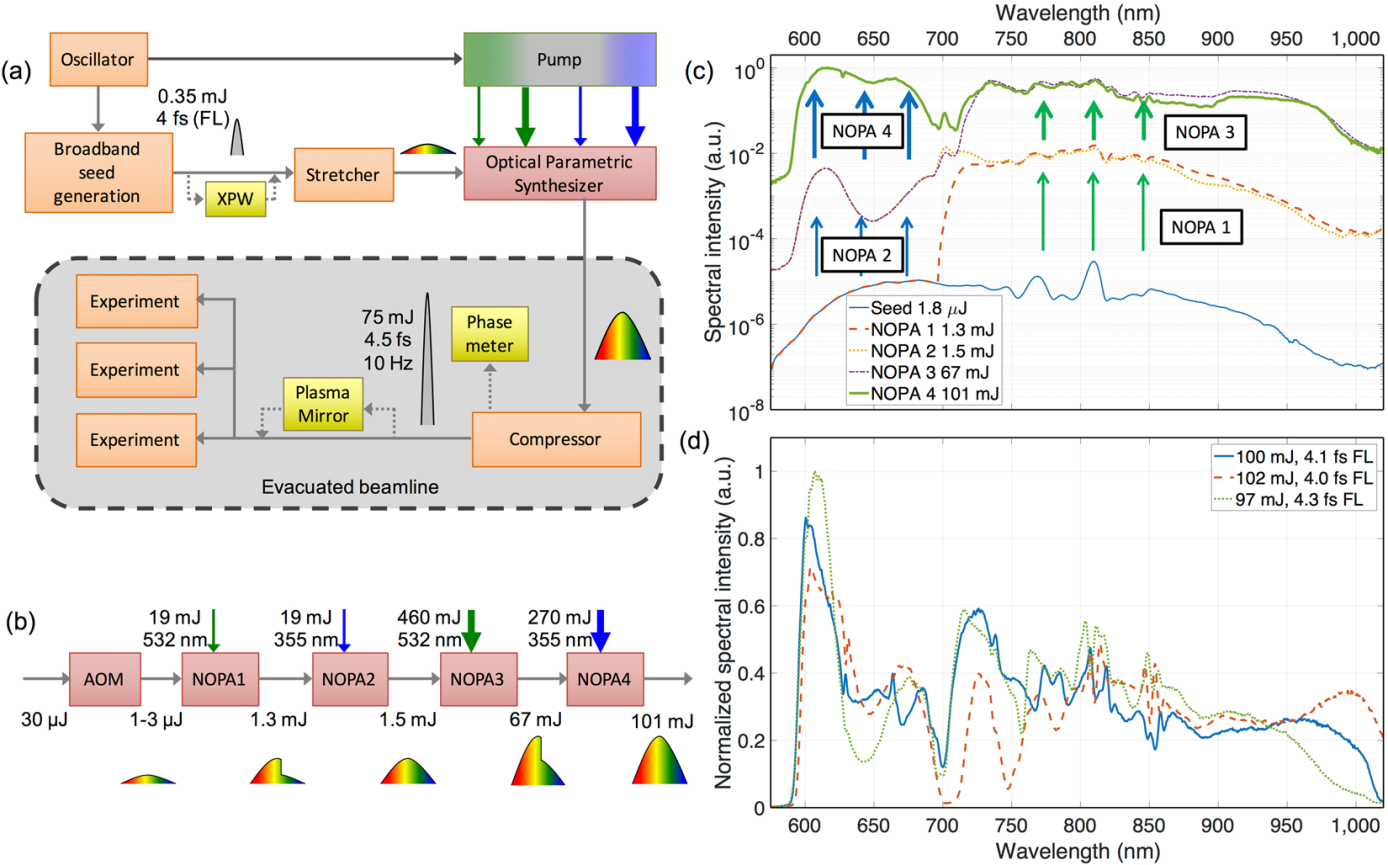
Working principles of the LWS-20 relativistic sub-two-cycle system. (**a**) Schematic of the light source. Pulses coming from a common broadband Ti:Sa oscillator are divided into a seed and a pump arm. The seed is generated by first amplifying the oscillator pulses in a 9-pass CPA system and then broadening them in a neon-filled HCF. After broadening, an XPW setup is optionally used for temporal contrast enhancement. The seed is then stretched in a grism stretcher and afterwards sent into the OPS. The pump for the four NOPA stages is generated in a Nd:YAG laser amplifier, providing a low-energy and a high-energy arm at both the second harmonic (532 nm) and the third harmonic (355 nm). After the OPS, the pulses are compressed and sent to the experimental chambers. A single-shot CEP meter is optionally used for electric-field-sensitive experiments. (**b**) Schematic of the OPS, depicting the temporal evolution of the pulses at the different amplification stages. The AOM first shapes the spectral amplitude and phase of the seed pulses which are then fed into the four NOPA stages. The first two NOPA stages are pumped by the two low-energy pump beams at 532 nm and 355 nm, respectively, and the latter two are pumped by the two high-energy pump beams. (**c**) Spectral intensity evolution in the synthesizer. Optical spectrum of seed (blue line), and after the first (orange dashed line), second (yellow dotted line), third (purple dash-dotted line), and fourth (green thick line) amplification stage. Consecutive energy enhancements in the visible and near-infrared parts of the seed provide a broadband spectrum beyond what is achievable in a single stage. (**d**) Spectral intensity after amplification, but before compression, for different configuration of the NOPA stages depicting the tuning capabilities of the OPS. The corresponding Fourier-limited pulse durations are indicated in the legend.

The LWS-20 is composed of the following sections: the oscillator, the broadband seed generation arm, the stretcher, the pump laser, the OPS, and the compressor as shown in Fig. 1a. The system is served by a broadband Ti:Sa oscillator (Rainbow HP, Femtolasers), as a common front end for pump and seed generation for the NOPA stages, ensuring optical synchronization between them. The oscillator produces pulses with an energy of 5 nJ and a bandwidth supporting 5.5 fs (full width at half maximum, FWHM) duration in intensity. The beam is split towards the seed and pump arm with an energy ratio of 60:40 respectively.

The seed generation arm first amplifies the pulses in a 9-pass CPA amplifier with a repetition rate of 1 kHz (Femtopower Compact Pro, Femtolasers). It provides pulses with an energy of 1 mJ and a FWHM pulse duration as short as 20 fs. 800 μJ from these pulses are broadened in a neon-filled HCF, reaching a bandwidth that supports 4 fs FWHM with pulse energies around 350 µJ. A cross-polarized wave generation (XPW) setup is optionally used after the HCF in order to clean the pulse contrast (see methods)^45–47^.

The broadband pulses are then temporally stretched for amplification, with their shortest- and longest-wavelength components separated by a group delay of 100 ps. It is accomplished by using a specially-designed grism stretcher similar to that described in^48^. It provides negative group-delay dispersion (GDD) and precisely-tailored higher-order spectral phase for stretching, in order to temporally compress at the end of the system with anti-reflection-coated bulk material. This bulk compressor introduces a positive GDD with a high throughput, maximizing the available energy for experiments. After stretching, approximately an energy of 30 µJ is seeded into the synthesizer, with a spectrum spanning over 580–1020 nm.

The other replica of the split oscillator output is seeded into a CPA-less (no stretching and compression) Nd:YAG pump laser (EKSPLA) operating at a 10 Hz repetition rate and incorporating second-harmonic and third-harmonic generation units of the fundamental wavelength of 1064 nm. The pump laser provides two separate arms at 532 nm and 355 nm, which are subsequently divided to pump the NOPA stages of the two OPS channels (see methods).

The synthesizer is composed of five sequential units: a custom-made programmable acousto-optic phase and amplitude modulator (AOM; Dazzler, Fastlite) and four NOPA stages. The modulator allows the spectral phase and amplitude of the broadband seed pulse to be controlled between 580–1020 nm. As it has a transmission of around 10%, approximately 2 µJ of seed reaches the NOPA stages. The pulses are transmitted at a 10 Hz repetition rate and due to dispersion inside the modulator crystal they are partially compressed to a duration of 65 ps.

The OPS is sketched in Fig. 1b,c (the NOPA stages are detailed in the methods). Briefly, the first and third NOPA stages are pumped with the 532-nm beams and optimized for amplification between 700–1020 nm, while the second and fourth stages are pumped with the 355-nm beams and amplify radiation between 580–700 nm. The first stage boosts the seed energy from the microjoule level to above 1 mJ and 0.2–0.3 mJ are added in the second stage, extending the bandwidth to the whole spectral range. The third and fourth stages are saturated at the energy levels of 60–70 mJ and 90–100 mJ, respectively. The amplified pulses exhibit energy fluctuation of less than 2% (rms) and a spectral distribution supporting durations down to 4.0–4.5 fs, see Fig. 1d.

## Spatio-temporal compression

The compressor is composed of a bulk glass and a set of chirped multilayer mirrors. At the output of the OPS the beam is expanded to 120 mm in diameter to suppress nonlinear effects during propagation through the bulk material. The amplified beam is compressed from ≈ 65 ps to ≈ 0.4 ps after propagating through 160 mm of SF57 and 100 mm of quartz. The beam size is then reduced to 50 mm in diameter and sent to an adaptive mirror, whose plane is imaged onto a Shack-Hartmann wavefront sensor. This setup corrects wavefront aberrations in a closed-loop configuration and yields an RMS wavefront error of 40–50 nm and a Strehl ratio of 0.9, which permits diffraction-limited focusing. Reflected from the adaptive mirror, the beam is sent into a vacuum chamber for additional compression through the reflection on four chirped mirrors, which add 75 fs^2^ of GDD each^49^.

After compression, the beam is sent into different experimental vacuum chambers through an evacuated beamline. At the chambers the spectral phase of the pulses is characterized and optimized through the chirp-scan technique^50^. Because of its simplicity, this method is ideal to measure the spectral phase on target, assuring optimally-compressed pulses for the experiments. This technique is implemented through the use of the Dazzler to scan the chirp (GDD), and a 5-µm-thick type-I BBO crystal to generate the second harmonic of the fundamental laser frequency. After optimization the spectrum on target becomes slightly narrower and the overall Dazzler transmission is reduced (see Supplementary Discussion). Compressed pulse energies between 70–75 mJ are reached in the compressor chamber, which corresponds to a factor of 30–100 compared to the state of the art^13, 51^.

**Figure 2:**
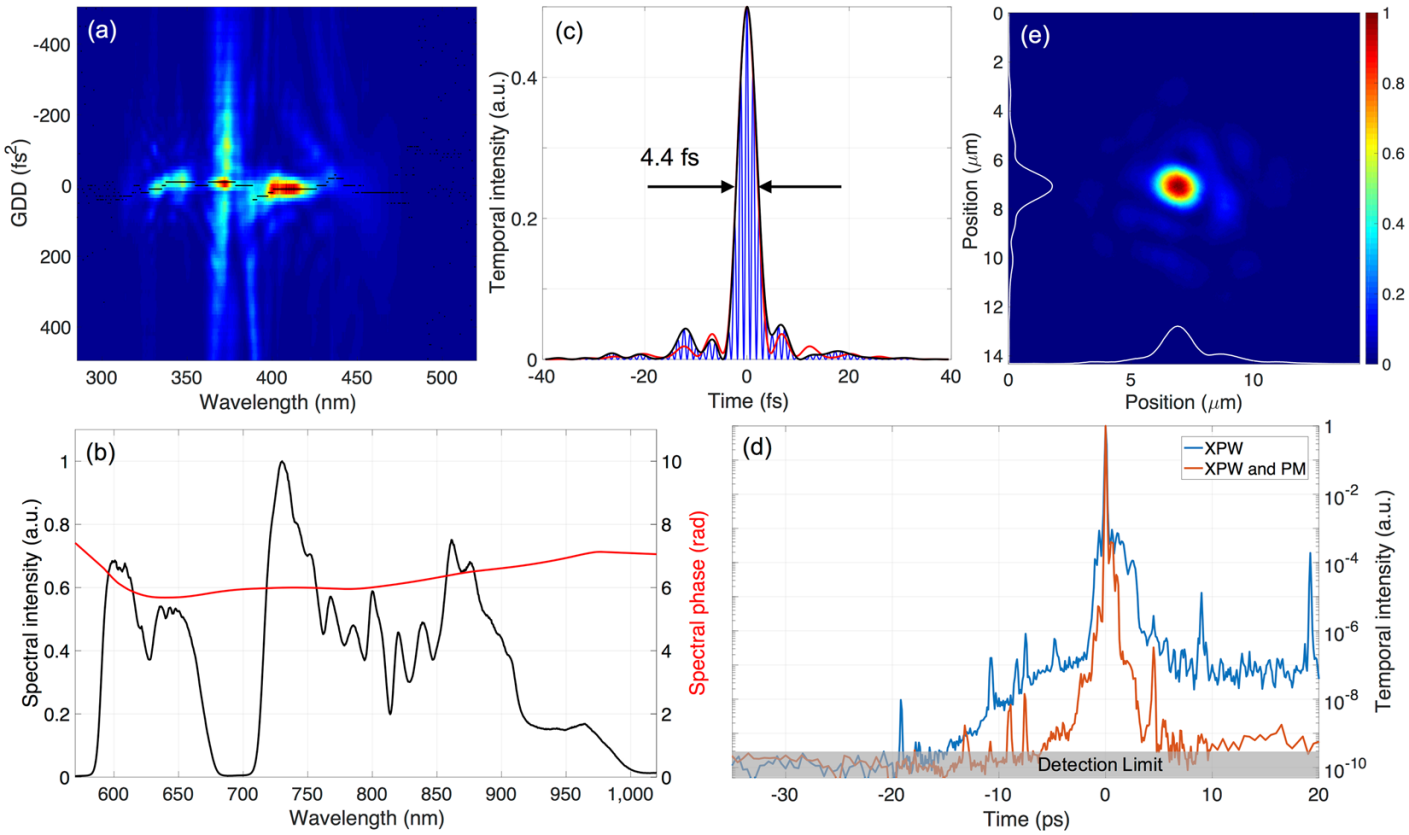
Spatio-temporal characterization. (**a**) Chirp-scan trace measured at one of the target experimental chambers, showing the second-harmonic spectral intensity as a function of wavelength and introduced GDD. The black curve shows the maximum harmonic intensity for each wavelength. (**b**) Retrieved spectral phase (red curve) and measured spectral intensity (black curve). (**c**) Temporal intensity (black curve), Fourier-limited intensity (red curve) and instantaneous intensity (blue curve) calculated from the chirp-scan retrieval. Both the evaluated and Fourier-limited durations are 4.4 fs. (**d**) Third-harmonic cross-correlation measurement of the temporal intensity with only XPW in use (blue dashed curve) or both XPW and the plasma mirror (PM) applied (orange solid curve). The detection limit of the device is approximately 10^−10^. (**e**) Single shot image of the focus, obtained with a 60-mm-focal length off-axis parabolic mirror showing a FWHM diameter of 1.3 µm, corresponding to a peak intensity of 1.3 × 10^20^ W/cm^2^.

The measured phase supports almost Fourier-limited pulses with typical FWHM durations of 4.3–4.5 fs, see Fig. 2a–c. This performance is additionally confirmed through the single-shot frequency-resolved optical gating (FROG) technique (see Supplementary Fig. S1) and routinely reproduced on a day-to-day basis. Furthermore, single-shot second-harmonic autocorrelation (SHAC) is also applied as an alternative temporal characterization technique, which provides information about the shot-to-shot pulse duration stability. The autocorrelator works at the repetition rate of the laser and shows an RMS error of 0.1 fs in the pulse duration. This proves the stability of the coherent combination of the two spectral parts and the robustness of the sequential OPS principle.

To achieve the high intensities required for ultra-relativistic laser-plasma investigations, the LWS-20 output is tightly focused by an off-axis parabolic mirror with a focal length of 60 mm, corresponding to an f-number of f_#_ = 1.2. Additionally, any further wave-front aberrations introduced between the adaptive mirror and the experimental setup are compensated, leading a focused spot diameter of approximately 1.3 µm FWHM, see Fig. 2d, containing 31% energy within the spatial FWHM. With these parameters, peak intensities about 1.3 × 10^20^ W/cm^2^ are reached, which corresponds to a normalized vector potential of a_0_ = 7.2. This intensity significantly exceeds the relativistic limit for electrons (corresponding to a_0_ = 1, i.e., 2.5 × 10^18^ W/cm^2^) opening a new realm of applications for intense few-cycle pulses. For experiments with overdense plasmas in this regime the temporal contrast plays a crucial role. A not sufficiently high contrast on the nano- to picosecond-scale (minimum 8–10 orders of magnitude below the pulse peak intensity, depending on the experiment) tends to generate a pre-plasma and is likely to deteriorate the interaction.

The contrast is characterized using a home-made third-harmonic cross-correlator^52^. In order to reach a contrast level of better than 10^–8^ several steps are taken^53^ (see methods). Due to the limited temporal amplification window, the NOPA intrinsically offers a contrast enhancement of 10^−5^. To further improve on this, the XPW setup located after the broadband seed generation arm is optionally used. However, its use reduces the available seed for the synthesizer, leading to a decreased pulse energy of 80–90 mJ after the fourth NOPA stage and a slightly narrower spectrum, still allowing sub-5-fs durations.

When XPW is in use, the contrast before the main pulse reaches 10^−7^ at t = −3 ps and 10^−10^ at t = −20 ps, see Fig. 2d, though there is still a prepulse reaching up to 10^−6^. The prepulse reaching up to 10^−8^ at t = −19 ps is a measurement artefact and is not generated from a postpulse during the amplification process, as reported in^54^. For additional improvement a plasma mirror supporting 10 Hz repetition rate is optionally used after the chirped mirrors^55^. It has a throughput of 80% and a typical contrast enhancement of 2.5 orders of magnitude, which leads to a value of 3 × 10^−8^ already at t = −1.5 ps.

**Figure 3:**
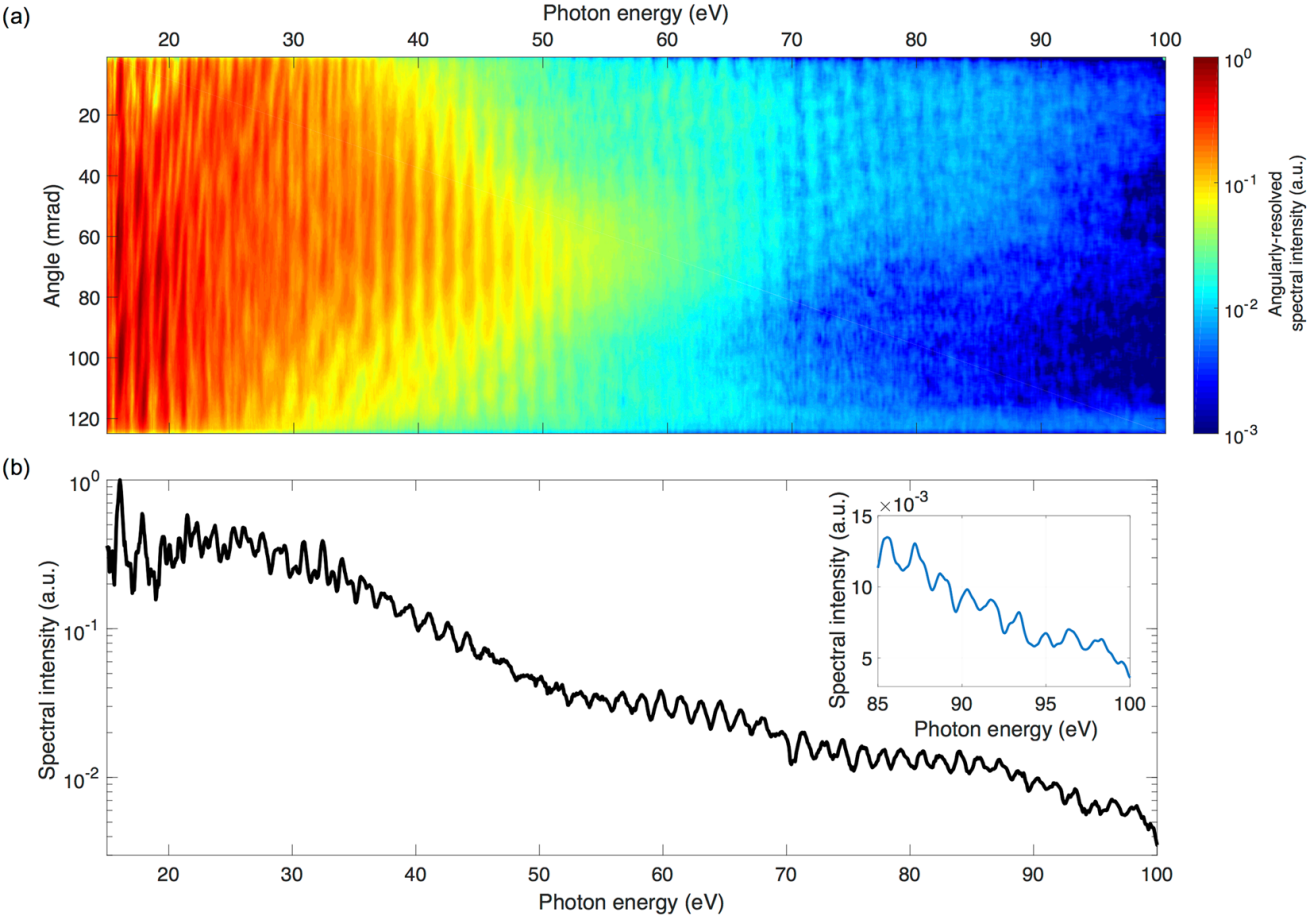
Relativistic high-order harmonic spectrum. The sub-5-fs pulse is tightly focused [see Fig. 2 (e)] onto a glass target and the reflected beam is directed into an XUV spectrometer. (**a**) Single-shot angularly-resolved high-harmonic spectral intensity of the generated XUV radiation in a logarithmic colour scale. (**b**) Corresponding angular integration between 0–20 mrad of the spectral intensity showing photon energy cutoff up to 100 eV. Inset: high-order harmonics at the highest photon energies in a linear scale.

As a demonstration of the applicability of the source in laser-plasma interactions in the relativistic regime, the temporally compressed beam is tightly focused onto the surface of a glass target and the emitted XUV radiation is spectrally and angularly characterized (see methods). The observed harmonic spectrum is shown in Fig. 3 and extends to a photon energy of 100 eV confirming the ultra-relativistic laser intensity and a satisfactory contrast. This would enable the generation of intense isolated XUV and x-ray attosecond pulses and electron bunches via relativistic interactions.

## CEP tagging

To study electric-field-sensitive processes an accurate control or measurement of the CEP is needed^4, 56^. For this purpose, a stereo above-threshold ionization phase meter was developed and implemented in the system^57^. This device allows the CEP of each shot to be measured, which is then correlated to the physical observables of the interaction under scrutiny for each individual shot (CEP-tagging, see methods).

**Figure 4:**
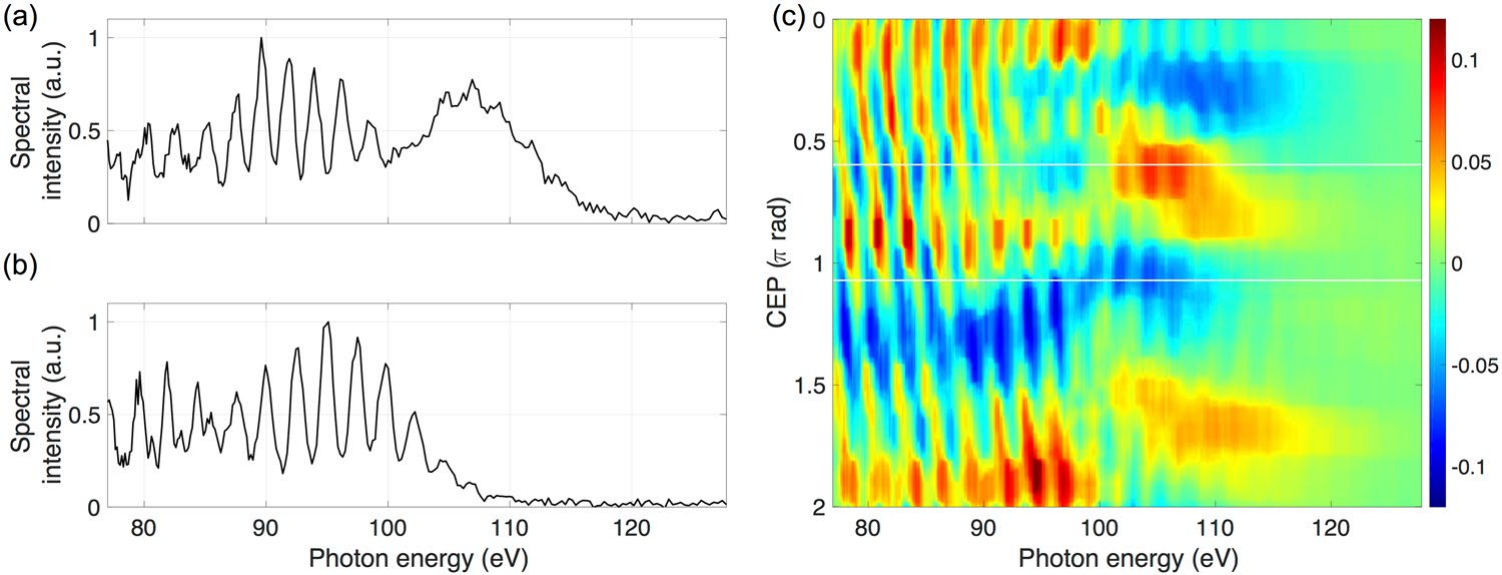
CEP-tagging demonstration. (**a,b**) Two single-shot XUV spectra at a relative CEP difference of π/2. The interference pattern in the spectral region above 100 eV disappears for (**a**). (**c**) Difference between spectral intensity and CEP-averaged spectrum, as a function of CEP. In the region below 100 eV a harmonic frequency shift is visible, corresponding to two complete harmonics within a 2π change of the laser’s CEP. In the region above 100 eV, changes in the spectral intensity with respect to CEP are observed to have a π-periodicity. The white lines mark the two single-shots shown in (**a**).

For testing the operation and utility of the phase tagging technique, high-order harmonics of the fundamental frequency are generated. To this end, the sub-5-fs, multi-terawatt pulses are gently focused (f_#_ = 300 approximately) into a 1.5-mm long neon gas medium, and the generated XUV spectra are recorded in a single-shot manner (see methods). This process is chosen due to the well-known CEP effects observed in XUV spectra arising from HHG^58^. Two single-shot spectra at a relative CEP difference of π/2 are plotted in Fig. 4a,b, exhibiting distinctly different patterns: (a) an almost 20-eV-broad continuum around 100 eV photon energy and a larger cutoff, or (b) a modulated shape and lower cutoff. These are significant indications of the expected electric field dependence of the process, which additionally confirm the short driving pulse duration.

To investigate this in more detail, 1000 shots are recorded and then ordered by the measured CEP, which are homogeneously distributed between 0–2π. For a better visibility, the CEP-averaged spectral intensity is subtracted for each shot, as shown in Fig. 4c. In the region below 100 eV a harmonic frequency shift is visible corresponding to two complete harmonics within a 2π change of the laser’s CEP phase. A similar periodic behaviour is observable also in the region above 100 eV, where the cutoff-region intensity changes with a π-periodicity. The observation of these well known effects demonstrates the feasibility of measuring electric-field-dependent effects with the system. This CEP-tagging can easily be extended for experiments at relativistic intensities as well.

## Conclusions and outlook

We have presented a novel optical parametric synthesizer closing the gap between low-energy few-cycle laser systems and high-energy lasers with pulse lengths of several optical cycles. It produces ultra-relativistic intensities with excellent temporal structure and sub-two-cycle duration. This unique combination of features is precisely characterized and even confirmed in two separate experiments. First, the short duration and ability to measure electric-field-dependent phenomena are supported via CEP tagging of high-harmonic and broad continuum generation in an atomic gas. Second, the high intensity and good temporal contrast are demonstrated by generating relativistic high harmonics up to a photon energy of 100 eV.

Optical parametric synthesis of tailored few-fs pulses is a versatile concept and scalable towards peak powers of the order of 100 TW and beyond as well as repetition rates of multi-kHz. The latter is based on the recent availability of kW-class picosecond pump sources^59^. These characteristics open novel opportunities in attosecond and relativistic laser-plasma physics. For instance, the generation of isolated relativistic electron bunches^21^ and intense XUV/X-ray pulses^26^ down to the few-attosecond or even sub-attosecond regime are within reach. The resultant laser-driven intense isolated attosecond pulses, will enable XUV/X-ray pump-probe spectroscopy of core electron dynamics, a research field considered only with free-electron lasers.

## Methods

### Pump laser

The pump portion of the oscillator output is first sent into a photonic-crystal fibre which generates approximately 20 pJ of energy behind a 10-nm FWHM filter centred at 1064 nm^60^. The resulting pulses are amplified to 2.5 nJ in a fibre amplifier (Keopsys) from which approximately 2.5 pJ is contained within the pump laser bandwidth. These are then seeded to the Nd:YAG-based laser amplifier. The pulses are first amplified in a diode-pumped regenerative amplifier to approximately 2 mJ and then divided into two arms. Each of the arms include a double-pass amplifier and two single-pass amplifiers, all flash-lamp pumped. The two parallel arms of the pump laser generate high-quality pulses of 1 J of energy each at a 10 Hz repetition rate and at 1064 nm, corresponding to the Nd:YAG central wavelength. The pulses have an RMS energy stability of less than 0.4%, a FWHM duration of 80 ps and a tenth order supergaussian beam profile. One arm is frequency doubled and the other tripled, respectively reaching up to 550 mJ at 532 nm and 430 mJ at 355 nm. Both of these arms are further split into a smaller (5% /7.5% of the 532/355 nm energy respectively) and a larger energy arm and later relay imaged onto the four NOPA stages. The pump beams reach the first and second stages with a pulse energy of 19 mJ each, which corresponds to a peak intensity of 8.9 GW/cm^2^ and 5.7 GW/cm^2^ respectively. The pump pulses for the third stage have an energy of 460 mJ, reaching an intensity of 8.6 GW/cm^2^ and the pulses of the fourth stage have 270 mJ, leading to a peak intensity of 3.5 GW/cm^2^.

### NOPA stages

Each amplification stage consists of a type-I BBO nonlinear optical crystal, with a length of 4.5 mm for the first two stages, and a length of 5 mm for the second two. For the first and third stages, the crystals have a cut angle of 24° and a non-collinear angle of 2.23° between pump and seed. Similarly, the crystals at the second and fourth stage have a cut angle of 34.5° and a non-collinear angle of 3.40°. These angles are slightly tunable, which provides tailoring of the spectral gain between a continuous spectrum supporting longer pulses or shorter pulses with a discontinuity around 700 nm in the spectrum (see Fig. 1d).

### Contrast enhancement

The temporal contrast is defined as the ratio of the instantaneous intensity at a certain time to the overall peak intensity. Its enhancement through XPW relies on the third-order nonlinearity of the process. After the HCF the spectral phase of the pulses is pre-compressed through chirped mirrors and then sent through a polarizer to ensure a linear polarization. The pulses with a duration is approximately 5 fs are then focused into a 2 mm BaF_2_ crystal with holographic cut in the [011] direction^61^. In the crystal, the polarization of the peak of the pulse is rotated while the less intense background is unaffected. A second polarizer filters out the original polarization containing the low intensity background, thus transmitting a higher-contrast pulse which is recollimated and sent to the stretcher. The overall efficiency of the process is approximately 7%.

The additional contrast enhancement through the plasma mirror also relies on the intensity dependence of its reflectivity. Already in vacuum at the end of the system, the compressed pulses are focused with an off-axis parabolic mirror onto an anti-reflection coated glass target. The lower intensity portion coming before the main pulse is transmitted through the target. When the ionization threshold is reached a high-density overcritical plasma is generated. This reflects the main peak of the pulse, which now exhibits a higher contrast. The target is mounted on a rotation stage which provides an undamaged surface for each pulse. An approximate peak fluence of 10^4^ J/cm^2^ is used on the glass target, which indicates an already very high input contrast^62^. Approximately 80% of the energy is reflected by the plasma mirror, while the anti-reflection coating reflects <0.5%.

### Relativistic harmonics generation setup

The optional plasma mirror was prepared, but not operated in this experiment. The compressed pulses are focused with a 60 mm focal length, off-axis, silver-coated, parabolic mirror onto the surface of a fused silica circular plate, at a 45° angle of incidence. The plate is mounted on rotation and translation stages, which are synchronized with the laser such that each shot reaches an undamaged spot. Another translation stage shifts the target along the focusing direction. The XUV radiation is characterized with a home-made single-shot flat-field spectrometer. A 1200 line/mm grating (Hitachi) maps the spectrum onto a detector consisting of a combination of a multi-channel plate and a phosphor screen in vacuum-vacuum configuration. The spectrometer is calibrated using the oxygen emission lines at the spectral region between 15–100 eV.

### CEP meter

For measuring the CEP phase, a few hundred µJ of energy are split off the compressed linearly polarized beam by means of a pellicle beam-splitter placed at Brewster-angle. A 5-mm-wide silver-coated circular area in the middle of the pellicle reflects a portion of the beam that is then focused into the xenon-filled chamber of the phase meter. The laser pulse ionizes the gas, accelerating electrons along its polarization directions. The electron spectra are measured in a single-shot manner by two multi-channel plate detectors positioned opposite to each other along the electron beam path. The asymmetry between the two measured spectra at the highest electron energies can be related to the CEP of each shot, which is then used for tagging.

### Gas harmonics generation setup

For the phase-tagging demonstration experiment, the central portion of the laser is focused with an 8 m focal length spherical mirror onto a neon gas target. The on-target intensity is estimated to be 6 × 10^14^ W/cm^2^ from the XUV spectra cutoff photon energy. This intensity is below the saturation intensity for neon, thus allowing a clear observation of the known CEP effects on the high-harmonic spectra. The neon gas is delivered through a supersonic de Laval nozzle with a 1.5 mm aperture^63^. The pulsed nozzle provides a 1.5 mm long flat-top gas density profile.

After generation, the XUV and laser radiation travel collinearly onto a 400 nm zirconium filter located 12 m away, which only transmits photon energies above 60 eV. The XUV radiation is then detected with a home-made single-shot XUV flat-field spectrometer, similar to the one used for the relativistic harmonics experiment, but with an XUV camera (Andor, Newton) as the detector. The nozzle, spectrometer and phase-meter are synchronized and operated at the repetition rate of the laser.

## Electronic supplementary material

Supplementary Information
