# Supplementary material for: Next Generation Driver for Attosecond and Laser-plasma Physics
Source: Sci Rep. 2017 Jul 12;7:5224. doi: 10.1038/s41598-017-05082-w (PMC5507917; doi:10.1038/s41598-017-05082-w)
Supplement: Supplementary file 1 — Supplementary Information [file 41598_2017_5082_MOESM1_ESM.pdf]

Next Generation Driver for Attosecond and Laser-plasma Physics

Supplementary Information

D. E. Rivas,* A. Borot, D. E. Cardenas, G. Marcus, X. Gu, D. Herrmann, J. Xu, J. Tan, D. Kormin, G. Ma, W. Dallari, G. D. Tsakiris, I. B. Földes, S.-w. Chou, M. Weidman, B. Bergues, T. Wittmann, H. Schröder, P. Tzallas, D. Charalambidis, O. Raszkazovskaya, V. Pervak, F. Krausz, L. Veisz,*

*Correspondence to: daniel.rivas@icfo.eu, laszlo.veisz@umu.se

Supplementary Figure

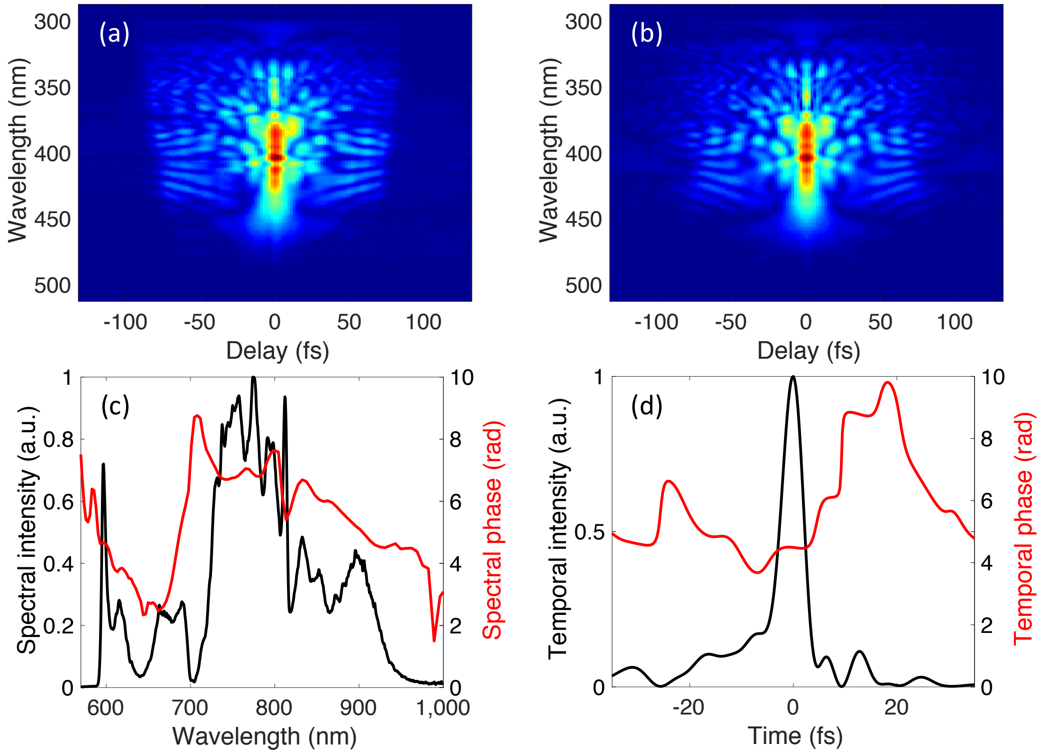

Supplementary Figure S1. FROG retrieval results (1). (a) Measured single-shot spectrogram. (b) Reconstructed spectrogram, with an error of 0.9%. (c) Retrieved spectral phase and measured spectral intensity at the FROG setup position. The spectrum allows a Fourier-limited pulse duration of 4.94 fs. (d) Calculated temporal intensity and phase from the retrieved spectral phase and measured spectrum, showing a pulse duration of 4.96 fs. Unfortunately, the optics delivering the pulses to the FROG setup reduce the spectral amplitude below 700 nm, which leads to a longer Fourier limit. Nevertheless, the retrieved pulses are still sub-two cycles, as confirmed also by the other methods. The optics delivering the pulses to the experiments support the whole spectral range.

Supplementary Discussion

AOM's spectral transmission

The AOM's transmission changes depending on the phase it is set to compensate (2). When allowing for a constant and high transmission used as reference from day-to-day, spectra supporting 4.0 fs are reached, with an energy of up to 100 mJ [see fig. 1 (d)]. After retrieving the residual spectral phase of the pulses in the target experimental chambers, the modulator is set to compensate for it. This results in a reduced energy transmission, particularly at the shorter wavelengths. The overall amplified energy is reduced by approximately 10% and the spectral bandwidth is decreased, leading to a Fourier-limited pulse duration of approximately 4.3 fs [see fig. 2 (d)]. After propagation through the compressor and phase compensation, pulse energies up to 75 mJ are reached, with a measured pulse duration down to 4.3 fs.

Supplementary References

1. Baltuška, A., Pschenichnikov, M. S. & Wiersma, D. A. Amplitude and phase characterization of 4.5-fs pulses by frequency-resolved optical gating. *Opt. Lett.* **23**, 1474-1476 (1998).
2. Kaplan, D. and Tournois, P. Theory and performance of the acousto optic programmable dispersive filter used for femtosecond laser pulse shaping. *J. Phys. IV France* **12**, 69-75 (2002).
